# The Importance of Functional Tests in Personalized Medicine

**DOI:** 10.5041/RMMJ.10114

**Published:** 2013-04-30

**Authors:** R. Jay Widmer, Amir Lerman

**Affiliations:** Division of Cardiovascular Diseases, Department of Internal Medicine, Mayo Clinic and College of Medicine, Rochester, MN, USA

**Keywords:** Cardiovascular disease, endothelial function, individualized medicine

## Abstract

Cardiovascular disease is the most prevalent disease mainly in the Western society and becoming the leading cause of death worldwide. Standard methods by which healthcare providers screen for cardiovascular disease have only minimally reduced the burden of disease while exponentially increasing costs. As such, more specific and individualized methods for functionally assessing cardiovascular threats are needed to identify properly those at greatest risk, and appropriately treat these patients so as to avoid a fate such as heart attack, stroke, or death. Currently, endothelial function testing—in both the coronary and peripheral circulation—is well established as being associated with the disease process and future cardiovascular events. Improving such testing can lead to a reduction in the risk of future events. Combining this functional assessment of vascular fitness with other, more personalized, testing methods should serve to identify those at the greatest risk of cardiovascular disease earlier and subsequently reduce the affliction of such diseases worldwide.

## PERSONALIZED MEDICINE AND HEART DISEASE

One of the goals of personalized medicine is to identify patients at risk for future cardiovascular events. Methods such as genomics, proteomics, metabolics, and transcriptomics are used to discern a marker or a set of markers that will identify the people who are at risk and also identify the optimal treatment for each individual. However, in certain areas, such as heart diseases, the predictability of these methods is lacking.

About 1.4 million heart attacks (myocardial infarctions (MI)) occur in the United States every year. The most common screening for heart disease is done by taking a history and conducting minimally invasive blood tests at the doctor’s office. These tests provide certain parameters such as blood pressure, cholesterol glucose, and C-reactive protein levels, which, as shown in the Framingham study,^1^ are the traditional risk factors for development of heart disease. For those falling into high-risk groups, non-invasive imaging such as angiograms can be performed; of the tens of millions of patients initially screened for coronary diseases, only a few million in the highest-risk group are in need of invasive imaging such as intravascular ultrasounds and thermographies.

Although this stepwise strategy is aimed at identifying those most likely to have significant coronary or arterial diseases, in reality the picture is quite different. One study has shown that of 136,905 people presenting with coronary diseases, 77% had normal low-density lipoprotein (LDL) levels.^2^ Another study has shown that 22.4% of patients who suffered an initial heart attack and took statins to aggressively lower their cholesterol levels suffered a repeat event (including death from any cause, myocardial infarction, documented unstable angina requiring rehospitalization, revascularization, or stroke) within 2 years despite having an LDL level of 62 mg/dL.^3^ An additional study has shown that the correlation between cholesterol levels and mortality from coronary diseases depends on geographical location.^4^ For example, in Japan, unlike the United States, little correlation was found between cholesterol levels and mortality rates. The risk of a Japanese individual having an MI was similar both for people who had higher cholesterol levels and for people who had lower cholesterol levels.

Four separate studies have shown that there is a relationship between stressful events such as earthquakes, missile attacks, stock market fluctuations, and even soccer matches and the incidence of acute coronary syndrome.^5^–^8^ Such events do not change cholesterol levels or the other classical risk factors, yet they may trigger an MI and sudden cardiac death. Even imaging the inside anatomy of the coronary arteries does not ensure predictability. After performing an angiogram on a group of patients, the predictability as to which patient would be the first to have an MI and where it would occur in the coronary arteries was low.^9^–^12^ Therefore, we must conclude that there are other risk factors involved in causing the onset of MIs, and we are missing functional tests that can pinpoint patients at risk for coronary heart diseases and sudden cardiac death.

## THE PREDICTIVE VALUE OF ENDOTHELIAL FUNCTION

Acute coronary syndrome and sudden death is a dynamic process. The process occurs at the interface between the vascular wall and the circulating blood. Thus, it is important to develop a test that measures vascular function, not one that just measures cholesterol levels or takes images of the arteries. If an intact blood vessel is put into a glass vessel and acetylcholine is added to the buffer, a relaxation of the blood vessel will occur. However, if there is an injury to the endothelial layer of cells or the blood vessel, this reaction will not happen. The main molecule that mediates this reaction is nitric oxide (NO).

Not all vessels or blood vessel segments react in a similar fashion. People whose blood vessels display abnormal endothelial-dependent reactivity have an increased risk for coronary diseases. Abnormal blood vessel reactivity was first measured in an experiment conducted 25 years ago. In that experiment, acetylcholine was infused into the left anterior descending artery. In some patients, the reaction to acetylcholine was normal, and the resulting effect was vasodilation. In other patients, the reaction to acetylcholine was abnormal, and the resulting effect was vasorestriction.^13^ As previously mentioned, not all people with high-risk factors will develop coronary diseases, while people with normal risk factors may go on to develop coronary diseases, suffer heart attacks, and even die from heart diseases. The reason for that phenomenon is that, in order to develop a disease, risk factors have to exert a negative effect on the vascular wall. They have to damage the vascular endothelium, which is not repaired, and this eventually leads to endothelial dysfunction or manifests as abnormal vascular reactivity. Such changes mediate the progression of plaque and hasten the event of a heart attack and sudden death. Indeed, both macrovascular endothelial dysfunction, as measured by flow-mediated dilation,^14^,^15^ and microvascular endothelial dysfunction^16^,^17^ have been found to be independent predictors of future cardiovascular events in large cohort studies in healthy individuals over and above traditional risk factor assessment. Endothelial function testing modalities have also been found to correlate with other novel cardiovascular testing modalities such as coronary calcium scoring.^18^,^19^

The endothelial layer responsible for the response to NO is also responsible for the body’s reaction to exercise and mental stress. In both these situations, the normal response of the arteries is endothelial deposit vasodilation, which increases blood flow to the myocardium. However, when vessels react abnormally, the blood flow to the myocardium is restricted, and the result is reduced oxygen supply.

## ENDOTHELIAL FUNCTION TESTS

The abnormally reacting endothelial layer is not limited to the coronary arteries but is a body-wide systemic reaction. This dysfunction is associated with other diseases such as stroke, vascular dementia, sleep apnea, and erectile dysfunction. However, the fact that this disorder is systemic can be advantageous because it allows detection through non-invasive diagnostic tests. If the endothelium reacts abnormally in the arm, finger, or leg, it can be used to identify a cardiac at-risk patient. Such a test was developed around 10 years ago and is based on the endothelium test to *reactive hyperemia*. In this test, blood flow is temporarily cut off using a blood pressure cuff. After the pressure is released, blood flow returns to normal after a short period of time. In normal people, a measurable dilation of the brachial diameter occurs at roughly 30 seconds following pressure release and tapers off at roughly 90 seconds. This response is not seen in people who have an endothelial layer dysfunction. The response of large blood vessels can be measured using ultrasound, and the response of smaller vessels, such as those in the finger, can be measured using an EndoPAT device (Itamar Medical Inc. Ltd, Caesarea, Israel).

Several studies have shown the predictability and efficacy of the endothelial function test. One study confirmed that endothelial dysfunction is associated with a higher rate of coronary adverse effects during a follow-up period.^15^ An additional study has shown that people with relatively normal risk factors but with endothelial dysfunction had a higher incidence of heart disease, hospitalization, and death after 5–6 years of follow-up as compared to those without endothelial dysfunction.^20^ If this parameter of endothelial dysfunction is added to the known Framingham risk score factors, we can better identify patients at risk for cardiovascular events. People with a high Framingham score and endothelial dysfunction are at the greatest risk, followed by those with a normal Framingham score but with endothelial dysfunction, and then those with a high Framingham score but with normal endothelial function. Least at risk are those with a normal Framingham score and normal endothelial function.^20^

## MENTAL STRESS AND ENDOTHELIAL FUNCTION

Mental stress is also mediated by endothelin. A difference in vascular response was seen between men and women who were put under mental stress.^21^ Normally reactive females and males behaved similarly, with an improvement in their blood flow after mental stress.

An example of stress-induced heart attacks can be seen in a syndrome called *apical ballooning*, or *takotsubo cardiomyopathy*, that affects mainly postmenopausal women. A study on women who had experienced stress-induced heart attacks showed that exposing them to mental stress caused their blood vessels to constrict instead of expand.^22^ This recognized functional link between mental stress and heart disease indicates that a susceptible group of people may be identified by using a functional test. Two additional studies have shown that when endothelial function was added to the known parameters that predict cardiovascular disease, the predictability of who would suffer coronary heart disease was substantially improved.^15^,^17^

## ENDOTHELIAL FUNCTION AND TREATMENT EFFICACY

In addition to the endothelial function test being a predictive parameter for coronary disease onset, it can also predict the effectiveness of a treatment given to patients with cardiovascular disease. One study followed a group of hypertensive women with no significant heart disease.^23^ All women received the same arterial hypertension treatment. After 6 months of treatment they underwent an endothelial function test. Both groups had a similar reduction in blood pressure. The group of women whose endothelial function improved had half as many cardiovascular events compared to those women who showed no improvement in endothelial function.^23^ In another study, patients with significant coronary diseases were treated with optimal medical therapy and given standard medications prescribed for their disease.^24^ At follow-up visits, the patients underwent endothelial function tests, and improvement was seen in 50% of the patients. As in the previous study, those with improved vascular response had fewer cardiovascular events during the follow-up period whereas the group that did not have improved endothelial function suffered from many more heart attacks during the follow-up period. Both studies showed that even after some of the markers were corrected the underlying disease remained.

According to a recent *Wall Street Journal* article (February 29, 2012),^25^ about 50% of males over the age of 65 take statins to lower their LDL levels. Statins are the most prescribed drug in the United States, with over 20 million Americans taking the drug. However, the FDA has recently issued a warning that prolonged use of statins can increase the risk of diabetes, stroke, and memory loss. Thus, by using endothelial function tests, we can guide administration of statins and other cardiovascular drugs, a more personal functional risk assessment approach.

## FUNCTIONAL TESTS FOR PREDICTING LESIONS

Novel imaging modalities have demonstrated that the arterial wall is not a homogeneous structure. There are areas that have no plaques, areas that have unstable plaque, and areas with stable plaque. In addition, not all blockages and lesions are created equally. Some lesions block the blood flow and others do not.

It is very important to differentiate between these stable types of plaque so that patients receive optimal care. The COURAGE trial (Clinical Outcomes Utilizing Revascularization and Aggressive Drug Evaluation) involved 2,287 patients who had stable coronary artery disease.^26^ Patients were randomly chosen to receive either percutaneous coronary interventions (PCI), such as stents and balloons, or optimal medical interventions, such as statins and beta-blockers. The study concluded that the trial showed little difference between invasive interventions (PCI) and medical interventions. However, no test was run to differentiate between the different types of plaque.

A subsequent study examined the COURAGE results and measured the fractional flow reserve (FFR) to differentiate between coronary lesions causing ischemia, and blockages not causing ischemia.^27^ The study showed that patients with ischemic blockages who were treated with surgical intervention had a long-term significant improvement in comparison with those who were only given medical intervention. In another multicenter study, patients with multi-vessel coronary artery diseases who underwent invasive interventions (PCI) with drug-eluting stents that were guided by FFR results had a significant reduction in the rate of the composite end-point of death, myocardial infarction, and target vessel revascularization at 1 year post-intervention.^28^ Another example of the efficacy of the FFR test can be seen in a study that showed an almost 43% potential reduction in the need for bypass surgery in patients who underwent FFR after having a coronary angiogram.^29^ It is therefore very important to examine the individual contribution to the physiology of an ischemic lesion, and not just its anatomy.

## FUNCTIONAL TESTS IN LARGE STUDIES

In the 1960s, a relatively small number of patients was sufficient to see a significant difference on cardiovascular events between treated and untreated groups. Today, however, since patients are getting better medical care and improved treatments, the delta between study groups is much smaller, thus necessitating studies of at least 20,000 patients in order to see significant differences between treatment groups. Therefore, it is of utmost importance to find ways to treat individual patients based on parameters such as individual functional tests so that true differences will be apparent without the need for very large and highly expensive mega-studies.

## CONCLUSION

In conclusion, functional tests have been found to be essential in predicting both at-risk populations and treatment outcomes. Using functional risk assessment tests can bring about an improvement in the individual’s health care outcome along with a reduction in health care costs. It is therefore just as important, and sometimes more important, to look at the functionomics of the individual patient and not only at the other four classical individualized healthcare parameters of genomics, proteomics, metabolics, and transcriptomics.

## Abbreviations:

FFR: fractional flow reserve;
LDL: low-density lipoprotein;
MI: myocardial infarction;
NO: nitric oxide;
PCI: percutaneous coronary interventions.
